# Poisons and Counter-Poisons Therapeutically Considered

**Published:** 1847-09

**Authors:** 


*Poisons and Counter-Poisons Therapeutically considered.* By M. Bouchardat.—Hôtel Dieu, in consequence of its central location, is the hospital most frequently selected by the administration and people of Paris whenever any accident or case of sudden disease occurs requiring immediate assistance. Among these there are of course a great many cases of poisoning, to which my attention has consequently been frequently directed. I have made also a great many experiments on animals in reference to counter-poisons, so that I think myself capable of giving some very useful information on this subject. I intend in this article to embody the results of such researches which have been as yet unpublished or scattered throughout my "Formulaire," my "Annuaires," the second edition of my "Manuel de Matière Médicale," or any other publications of the last year. I publish this notice in my "Annuaire" of 1847, because it is a work destined for the hands of both physicians and pharmaceutists, for I think that a thorough knowledge of certain parts of therapeutics is as necessary for the latter as the former; in fact, in urgent cases which call for immediate attention requiring a very exact knowledge of their nature, we are obliged to call upon the pharmaceutist, who is always at home, while the physician is very liable to be absent. In this respect there is a great deficit in the education of the pharmaceutist, which can only be remedied at present by publications like the one I here give.

The assistance required by any individual who has been poisoned may be considered under three heads. The poison having been discovered, the first indication is to evacuate it. For this purpose we employ emetics, emetics and cathartics together, purgatives, or the stomach-pump. The second indication is to administer the antidote. The third is to bestow upon the patient those attentions his condition requires, and which may be divided into those suitable in all cases of poisoning, and those adapted to each particular case.

1. To evacuate the stomach of its poison, tartar-emetic is most generally had recourse to; the dose is five centigrammes ($\frac{2}{3}$ of a grain) dissolved in half a tumbler full of water, and repeated two or three times after an interval of a few minutes. The patient should drink freely of tepid water, and it is often advantageous to assist the inclination to vomit by tickling the fauces. If tartar-emetic is not at hand, it may be substituted by the sulphate of copper, twenty centigrammes, (three grains,) dissolved in two table-spoonsful of water, and repeated if necessary; sometimes this emetic is preferable to the former because it acts more promptly.

When the poison is insoluble, and there is reason to believe that it has passed out of the stomach into the small intestines, it is better to administer an emetic and cathartic combined. Twenty centigrammes (three grains) of tartar-emetic may be dissolved with sixty grammes (two ounces) of sulphate of soda or magnesia in two pints of water, and then administered to the patient as rapidly as he can take it. It has been recommended in cases of poisoning from vegetable substances to use a strong solution of table salt, which acts both as an emetic and a cathartic, 50 grammes (an ounce and a half) to to the litre (two pints) of water. This is at times a very valuable therapeutic agent because it is always at hand, and we cannot administer an evacuant too soon.

When the poison has been taken per anum, and has entered the large intestines, we must have recourse to enemata. A very excellent enema may be prepared in these cases composed of twenty grammes (five drams) of senna, fifty grammes (an ounce and a half) of sulphate of soda and five hundred grammes (one pint) of water; this injection is much better than those more drastic in their action; these are slower in their operation, and I have often seen them prescribed without success. When we cannot succeed in procuring emesis, and the poison is still in the stomach, it is best to introduce the stomach tube and pump out the contents.

2. A counter-poison is, according to my view, any substance which will form an insoluble or inoffensive compound with the poison which has been taken. There are some general rules which it is necessary to mention in reference to the employment of these counter-poisons. We ought to give the preference to those which are perfectly innoxious, and which are most likely to be at hand. We should administer a quantity which will more than suffice to neutralize the poison, for the antidote may be rejected almost as soon as it is swallowed, and in the most fortunate cases even the compound is not wholly insoluble; then again we wish to destroy the poison as quickly as possible, and we can do this most readily by having the antidote in excess.

There are several counter-poisons which form compounds with the poisons which are possessed of a slight degree of solubility; these may after some time become redissolved in the intestinal canal, and cause a return of the bad symptoms. In these cases it is necessary to induce full evacuation of the stomach and bowels after administering the antidote.

When the poison has passed into the small intestines an insoluble counter-poison should be selected, otherwise its action might be limited to the stomach.

3. Poisoning is a disease produced by a known cause, and it is necessary to treat it with those remedies whose powers we have learned by experience. In almost if not quite all cases of poisoning, death results as a consequence of the great disturbance which takes place in the organs of respiration and circulation. It is necessary, therefore, to watch these organs, the constant exercise of whose functions is indispensable to life, and to take every means to prevent their suspension, for with their suspension comes death.

The circulation is to be kept up by sustaining the animal heat by means of warm clothing, frictions, hot water and sinapisms applied to various parts; sometimes it is well to practice moderate venesection. Respiration is assisted by the free admission of fresh air, by alternate pressure on the walls of the thorax, by blowing into the nostrils, or by galvanism. When the secreting organs eliminate readily any poison that may have been absorbed, it is important to increase the activity of such, as Orfila has recommended in advising the use of diuretics in poisoning from antimony and arsenic which are separated from the blood by the kidneys, and as we have recommended in prescribing those articles which increase the secretion of bile when the poison is separated by the liver;—this is the case with almost all the mineral poisons.

When the poison has been absorbed and cannot be easily and quickly removed from the economy, and if we cannot act upon it in the blood with the antidote, we must have recourse to remedies which are not injurious in themselves, and yet counteract the effects of the poison. This is the way that coffee acts in poisoning from opium.

We shall now commence the particular consideration of the individual poisons.

*Poisoning from Acids.*

I think I have explained in the most satisfactory manner the therapeutic effects of poisoning by acids.

In cases of poisoning from acids, the antidote alone is sufficient to restore the patient, without having recourse to evacuants.

All authors on therapeutics and toxicology recommend for neutralizing the acids the administration of magnesia, alkaline carbonates, soap, &c.; this is all very well, but it does not suffice. I think I have done a great service in founding a precise treatment to be pursued in these cases of poisoning.

In the first place (as recommended by every one) we must administer magnesia in excess; I prefer the hydrated magnesia, the preparation of which will be given in another place, in speaking of M. Bussy's work on an antidote for arsenious acid. This alkaline earth possesses some very great advantages; it is innoxious, purgative and insoluble, so that it can pass into the small intestines and neutralize any acid that may have passed into them; but its insolubility unfits it for fulfilling one very important indication. I have proved that in cases of poisoning by sulphuric acid, the acid is absorbed, and that when it gets into the blood it may cause clots to form which will interfere with the circulation and thereby cause death.* It is necessary, therefore, to follow up the sulphuric acid which has been absorbed, by giving after the magnesia some soluble alkaline substance which will be readily absorbed, and dissolve the clots which may be forming. The best alkali for this purpose is the bicarbonate of soda. It should not be given before the magnesia, because the large amount of carbonic acid which would be disengaged might facilitate those perforations of the stomach which are so much to be feared in poisoning by acids. Magnesia is not liable to this objection; and we must first neutralize with it the acids contained in the digestive organs. The following is the formula which I have often adopted in these cases of poisoning.

1. Twenty to fifty grammes (five to twelve drams) diffused in a litre (two pints) of water.

2. After the use of the magnesia, the patient must drink freely of a solution of bicarbonate of soda, ten grammes (two drams) to the litre being a good proportion for the solution.

Since I have been connected with the Hôtel Dieu, this treatment has been adopted at my request several times in very grave cases, and it has always been successful. It has been prescribed in cases of poisoning from sol. of indigo in sulphuric acid, from sulphuric acid diluted with its own weight of water, and from nitric acid.

N. B. It must be remembered that this treatment will not answer where the soda salts formed by the acid are poisonous, as is the case with arsenic acid, &c.

*Poisoning from Alkalies.*

The only treatment in these cases is that recommended by all authors. It is necessary to administer lemonade, or vinegar and water, in a sufficient quantity to neutralize the alkali which has been taken. If potassa or its carbonates have been taken, I prefer tartaric acid, for the potassa salts are not so innoxious as is generally supposed, and the bitartrate of potassa is the least objectionable of them.

*Poisoning from Arsenious Acid.*

Within a few years there have been a great many experiments made to discover which is the best antidote for arsenious acid, and

* In two cases, I, as well as C. Couriard, have found these clots lodged at the commencement of the femoral artery, in consequence of which the circulation in that leg had been stopped and mortification brought on, which caused the death of the individual.

the matter appeared to be entirely settled until some new views of M. Bussy have made very important additions to this subject.

M. Bussy has shown that magnesia, by forming an insoluble compound with arsenious acid, may be very advantageously employed in cases of poisoning by it;—M. H. Lepage has sent him a communication which goes to prove the efficacy of this new antidote. Although this may be the case, until the magnesia be well tested I should be unwilling to give up the hydrated sesquioxide of iron which M. Landras and I have thoroughly tested in various cases by experiments on animals.

If I were called to-day to render my assistance to a person poisoned by arsenious acid, this is the way in which I should act.

In the first place I should bring on emesis, according to the rules given above; then I should give the hydrated sesquioxide of iron diffused in two litres (four pints) of sweetened water. It is necessary to give this antidote in great excess, for, as M. Orfila has proved, if we give only what is actually necessary to form the arsenite of the peroxide of iron [properly, the subarseniate of the protoxide of iron] the power of the poison is not wholly destroyed.

If there is no hydrated sesquioxide of iron at hand, we must not hesitate to give *thirty grammes* (six drams) of the subcarbonate of iron, diffused in a litre of water while the former is being prepared. Experience has proved to us the great value of this course of treatment.

Our experiments on dogs have shown likewise that the persulphuret of iron in jelly is an antidote to the arsenious acid.*

I think it much better, however, at the same time, that we give the hydrated sesquioxide of iron, to administer twenty grammes (half an ounce) of magnesia. This must be a very valuable agent, not only because it forms an insoluble compound with the arsenious acid, but also because it purges and in that way follows up any arsenic that may have got into the intestines, and by increasing the number of evacuations it is likely to carry it off. To repeat then, I would give the hydrated sesquioxide of iron and magnesia both together; and as

* M. Mialhe has lately again strongly recommended the employment of the protosulphuret of iron in poisoning from arsenic, but in the experiments we have made, this article is much less efficacious than the hydrated sesquioxide of iron.

The persulphuret of iron is a very good antidote for several metallic substances; it acts like the protosulphuret to which M. Mialhe drew attention at the same time that M. Sandras and I were engaged in the experiments published in our article on the counter-poisons of arsenic, lead, mercury and copper, (Annuaire Thérapeutique, 1844.) We cannot admit with M. Mialhe the non-existence of the hydrated persulphuret of iron described by Berzelius: this compound is always formed when the alkaline persulphuret is in excess; but this is not the case when there is an excess of the persulphate of iron. We insist on this with M. Berzelius, and we have said that it was indispensable that the persulphuret of iron should be poured into the persulphuret of potassium.

We must observe also that the persulphuret of iron changes very rapidly when exposed to the air, sulphur being set free. This is the reason why we recommend it to be prepared with saccharine matter, and to be kept in well-stopped bottles.

this latter substance only acts when prepared in the manner given by M. Bussy, I shall give its preparation.

After administering the antidote, if the pulse is feeble and the skin cold, we must favor reaction by sinapisms, warm coverings, frictions, small bleedings, stimulating drinks, &c. When the cold stage is past and is followed by reaction, at the same time that we keep the bowels in an open state, we should give diuretic, nitrous drinks, as recommended by M. Orfila. In this way we will hasten the removal of the poison from the system, both by the bowels and by the kidneys.

*Preparation of the Magnesia employed as an Antidote for Arsenious Acid.*—(Bussy.)

Magnesia of the proper kind may easily be obtained in the following way: Fill a crucible half full of the carbonate of magnesia of the shops, then heat it until the bottom becomes of a dull red, and during the calcination the magnesia is to be constantly stirred with an iron spatula. When it is completely calcined the ebullition caused by the escape of the water and carbonic acid ceases, then a portion should be tested with muriatic acid, and if the calcination is complete there will be no escape of carbonic acid; but it is better not to perfectly calcine it than to expose it to too great a heat.

When the magnesia has been properly prepared it forms a hydrate without any difficulty; it makes with water at the ordinary temperature a consistent jelly, like alumina; two grammes of magnesia are sufficient to do this, with fifty grammes or more of water.

This quantity of magnesia diffused in a decilitre (three ounces) of water, is capable of combining, as we have said, with one decigramme (one and a half grains) of arsenious acid dissolved in a decilitre of water, so that after agitating them together for a moment and filtering, there will be no precipitate formed with sulphuretted hydrogen.

It is necessary to be careful not to use magnesia which has been too highly heated, or its effect will be almost entirely destroyed. Such magnesia is easily recognised in this way. Its density and its cohesion are greater than they should be, and instead of uniting with the water it falls to the bottom and forms a pulverulent deposit which may rest several months in contact with the water without becoming hydrated.

Hydrate of magnesia may also be prepared by precipitation, and this is very efficacious in cases of poisoning. 100 grammes of sulphate of magnesia in crystals contain 51.22 of water, 16.26 of magnesia, and 32.52 of sulphuric acid. It requires theoretically 38.21 of pure potassa, or 45.52 of the hydrate to decompose completely a solution of 100 grammes of the epsom salt and precipitate from it the magnesia in a hydrated form. But if instead of the pure potassa we take the ordinary caustic potassa, which always contains some chloride, sulphate, and carbonate, and an excess of water, it is best to take fifty for every hundred parts of salt to be decomposed. If the solutions are well diluted; if the sulphate of magnesia, for instance, is dissolved in twenty-five times its weight of water, and the potassa in twenty times its weight, it will not be necessary to wash the precipitate, but simply to express the excess of liquid between the folds of linen; the small quantity of sulphate of potassa and of sulphate of magnesia contained in it can have no injurious effect. For administration it is best to diffuse the precipitate in a large quantity of water. Ten grammes of sulphate of magnesia dissolved in 250 grammes of water, decomposed, as above directed, by five grammes of caustic potassa dissolved in 100 grammes of water, will still leave a liquid containing an excess of sulphate of magnesia, which might still be precipitated with potassa. The precipitate prepared according to the above proportions, when dried between the folds of linen without being washed, and then diffused in water, will combine immediately with one decigramme of arsenious acid dissolved in a decilitre of water. I may observe that this proportion of arsenious acid is not at all the maximum quantity the magnesia is capable of neutralizing. The calcined magnesia when properly prepared appears to me just as good, and is more readily made than the precipitated hydrate.

*Poisoning from Corrosive Sublimate and other Salts of Mercury.*

M. Orfila has discovered that albumen is an excellent antidote for corrosive sublimate; its efficacy has indeed been proved in a great many cases; it is a substance very frequently employed, and is in the hands of every one, while at the same time it is very safe.

The moment any symptoms indicating poisoning by mercury show themselves, the patient should take some yellows and whites of eggs mixed with water. Too much albumen must not be taken, for if it did not vomit it might redissolve the precipitate.

It will be well at the same time to make the patient swallow immediately, or as soon as it can be procured, 50 grammes of hydrated persulphuret of iron, or 10 grammes of Raciborski's iron, the efficacy of which has been proved by M. Sandras and myself in cases of poisoning by the salts of mercury.

It is indispensably necessary to favour emesis and purging by the free use of fluid, mucilaginous drinks. Cullerier saved two hundred patients who had taken over doses of corrosive sublimate, by making them each drink in the course of twenty-four hours seven or eight litres of milk, flaxseed tea and warm water.

*Poisoning from the Salts of Copper.*

The best counter-poison for the salts of copper, the certainty of which I together with M. Sandras have proved by experiments on animals, is the metallic iron reduced by hydrogen; it must be administered in a quantity at least as great as that of the poison which has been taken.

If the hydrate of the persulphuret of iron can be had, it may also be prescribed with a great deal of benefit. 100 grammes of the hydrate diffused in 200 grammes of sugar and water may be given.

If neither Raciborski's iron nor the hydrate of the persulphuret is to be had, albumen and water must be given, (six whites of egg mixed with a litre of water.) The albumen forms insoluble compounds with the salts of copper, as has been proved by Orfila in repeated experiments.

There has been a great deal of discussion on the utility of sugar in cases of poisoning from the salts of copper. The advocates of this substance maintain that the poison is decomposed by it. This explanation is erroneous; in fact, if the sugar, together with the gastric juice, is able to act at all on the salts of copper, it is at a higher degree of temperature than is ever found in the stomach. I know that persons poisoned by copper have been saved by being crammed with sugar, and by being vomited and purged at the same time, but the action of the sugar is different entirely from what they supposed. M. Sandras and I in our experiments on sugar as a nutriment, found that when it was given in excess, absorption was very considerably diminished, in accordance with the laws given by M. Dutrochet. It is easy to understand, therefore, how sugar may be useful while the poison is being removed by emetics and cathartics; but it cannot rank with counter-poisons, but with evacuants and substances which lessen absorption.

*Poisoning from the Salts of Lead.*

There are three things to be considered in the treatment of poisoning by the salts of lead.

1. The treatment of active poisoning by those salts taken in large doses and a small number of times.
2. The treatment of slow poisoning.
3. Prophylactic treatment.

I have already spoken of the two last heads in the second edition of my work on Materia Medica, and I have republished it in my "Annuaire" of 1846. I only give in this number the recipe for the syrup of the hydrate of the sulphuret of iron which M. Sandras and I have adopted, (page 101.)

It only remains for us to speak of the first head, or the active poisoning by lead.

The counter-poison on which the most reliance is to be placed, is the hydrate of the persulphuret of iron, which must be given to the patient in excess. It is best to administer the hydrate mixed with once or twice its weight of this syrup. If the hydrated sulphuret of iron cannot be had, 50 grammes of sulphate of soda or magnesia may be prescribed.

In both cases we may bring on vomiting and purging by the assistance of two drops of croton oil.

*Poisoning by Sulphuretted Hydrogen or by the Effluvia of Privies.*

In these cases of poisoning chlorine must be breathed by the patient, with caution, however. M. Labarraque saved a privy-cleaner who was asphyxied, by placing under his nose at intervals a sponge applied in a solution of chloride of soda. If the chloride of soda is not to be had, and all success depends on prompt action, we may get at any grocer's the "eau de Javelle," which is a chloride of potassa, with which a sponge should be wet and carried cautiously under the nose of the patient. The disengagement of chlorine may be increased at pleasure by pouring a few drops of vinegar on the sponge. Another way, recommended by M. Mialhe, for obtaining a slow and gradual disengagement of chlorine, is to enclose in a compress a handful of the chloride of lime, and to pour on it a few drops of vinegar.

When the patient begins to breathe he must have fresh air, and the circulation must be kept up by brushing the skin with a flesh-brush, and by wrapping him with warm coverings. Venesection should be practised, and then he should take an anti-spasmodic potion with two grammes of ether.

*Poisoning from the Liver of Sulphur, or its solution, known as the* "radical de Barèges, solution pour bains sulfureux, sulfure de potasse liquide," *(or by any alkaline sulphuret.)*

Vomiting must be obtained immediately in these cases by administering freely of tepid water, warm mucilaginous drinks, and by tickling the fauces; if these means do not cause emesis, the stomach-pump must be used. Neither the tartar emetic, nor the sulphate of copper or zinc can be used, because they are decomposed by the alkaline sulphurets. We administer afterwards, so long as the matters ejected have the odour of putrid eggs, the solution of ten grammes of the proto, or what is better, of the persulphate of iron, in one litre of water, with 200 grammes of sugar, in divided doses. Instead of the sulphuret of potassium, which is poisonous, we will have the sulphate of potassa formed, which is purgative, and the sulphuret of iron, which is insoluble. I have lately discovered and proved the efficacy of this antidote, which is very valuable, for the double decomposition is instantaneous, and the two compounds which result are innoxious; but as the sulphate of iron is poisonous of itself, we must watch its employment, and stop giving it as soon as the vomited matters contain no alkaline sulphuret.

### *Poisoning from Hydrocyanic Acid.*

1. There is no time for administering an emetic.
2. In poisoning by hydrocyanic acid the antidote must be administered immediately; for if death follows at all, it does so quickly.

When the acid is anhydrous, or even diluted with its own volume of water, it acts so quickly that M. Larroque thinks there are no hopes in administering the antidote; but if it is the medicinal acid, and especially if that has been diluted, the absorption is much more gradual, and we may hope for success in administering the antidote of M. Smith, which may be prepared beforehand, as follows, and which, according to M. Larroque, may be kept several months by the following precautions: To the solution of the two sulphates of iron we add a solution of sugar; we then precipitate with the carbonate of soda, and keep it in vessels filled and closely stopped.

| | | |
|---|---|---|
| Sugar, | - - - - - | 60 grammes. |
| Sulphate of protox. of iron, | - - | 55 " |
| Bisulph. of deutox. " | - - | 90 " |
| Water, | - - - - - | 250 " |
| Carb. of soda in crystals, | - - | 500 " |

Chlorine employed concurrently with the counter-poison of Dr. Smith may be of great service.

3. When the animal is apparently lifeless, free effusions of cold water on the spine should be made. I, as well as M. Louyet, have tested this plan, recommended by Dr. Robinson.

*Poisoning from Vegetable Alkalies and Substances which contain them.*

The largest proportion of the vegetable alkalies may be regarded as very dangerous poisons to man. They act in small doses and are very quickly absorbed. It is of the greatest importance, therefore, to evacuate the stomach of them as quickly as possible, according to the directions previously given.

The only counter-poison which has been as yet discovered, is the compound solution of iodine. I have given in my "Annuaire" of 1842 all the details of the insoluble compounds which result from the reaction between this preparation and the vegetable alkalies; they are ioduretted hydriodates, and are insoluble in acidulated water. Of all the insoluble compounds formed with a vegetable alkaline base, there is none which is so insoluble in acidulated water as this.

The following is the formula which I make use of for an antidote to vegetable alkalies:

| | | |
|---|---|---|
| Iodine, | - - - - - | 20 centigrammes. |
| Iodide of potassium, | - - | 40 " |
| Water, | - - - - - | 500 grammes, |

not 5 centigrammes, as has been printed (p. 33) in the second edition of my "Manuel de Matière Medicale."

This solution should be given in doses of half a tumbler full at a time, and vomiting should be kept up so that none of the precipitate shall remain behind.

This counter-poison has been used with success in cases of poisoning by opium, the salts of morphia, aconite, and I have tried it on animals poisoned by *opium*, the salts of *morphia*, *belladonna*, *stramonium*, *tobacco* and *strychnia*. It may be also used with success in poisoning by *cicuta*, *œnanthe* and other *umbelliferæ*, by *stavesacre*, *white hellebore*, *sabadilla*, *colchicum* and the salts of *quinine*, but it is of no use as an antidote to *digitalis*.

I cannot say whether it would be useful in cases of poisoning by the *agaricus*.

The compound solution of iodine is always more useful as a counter-poison for vegetable alkalies than the nut-galls or tannic acid; for although the latter forms an insoluble compound in almost all cases, it is very liable to be redissolved by an excess of acid, which is often found in the stomach.

*Poisoning by Opium and the Salts of Morphia.*

1. The stomach must be emptied by emetics, by cathartics, and, if possible, by the stomach-pump.

2. The compound solution of iodine should then be administered.

3. The narcotic effects should be counteracted by the use of coffee in large doses. This is not an antidote, but nevertheless it is an excellent remedy to counteract the narcotic effects. The best way is to administer it without sugar, but with the addition of a small quantity of alcohol. I give it thus because I wish it to be absorbed very quickly, and it is known that sugar retards absorption in the stomach. All our experiments on the digestion of sugar go to prove this fact; alcohol in small quantity favours absorption. The following is the recipe I make use of.

Coffee, roasted and ground, 50 grammes; from which we obtain by lixiviation, 500 grammes of the fluid extract; to this add 20 grammes of brandy, one-fourth of which is to be taken at intervals of five minutes. Coffee may also be administered by injection.

*Poisoning by Belladonna, Stramonium, Tobacco, and other Acro-Narcotics.*

1. Evacuate thoroughly the digestive organs.

2. Administer the compound solution of iodine, keeping up at the same time the vomiting.

3. Bleeding, and if reaction is pretty great, cooling drinks, baths, &c.

*Poisoning by Nux Vomica, St. Ignatius' Bean, Strychnia, Brucia and other products or compounds of Strychnia.*

Strychnia and its compounds does not give the least offence to the stomach, and it is for this reason that carnivorous animals, such as lions, wolves and dogs, whose stomachs are so sensitive that they reject all other poisons, may be readily destroyed by this substance.

1. It is best, therefore, to bring on full emesis as soon as possible; strong salt water or tartar emetic, are the agents to be employed in preference.

2. The compound solution of iodine should be given at the same time.

It is necessary to give an excess of the antidote, for I have proved that the resulting compound, although wholly insoluble in acidulated water, could still poison. It is true it requires a larger dose and a longer time for the poisoning to take place, but still it is best to give plentifully of the antidote and keep up free emesis.

3. We have as yet no good clinical reports on the means necessary to counteract the tetanic convulsions caused by the absorption of strychnia; there are, however, some general principles that may be followed without danger.

Death in these cases always results from asphyxia, because the respiratory muscles, whose constant action is so indispensable to life, become so rigid that they lose their functions. In this respect strychnia differs essentially from other poisons, the preparations of mercury for instance; the latter destroy vitality, while the former increases to a high degree the action of the involuntary muscles, which is the cause of the asphyxia. If the respiration could be continually kept up until the paroxysms had passed off, all danger, all disease would disappear, unless it be a lassitude following the intense excitement. This is not the case with other poisons; convalescence in those cases is slow, difficult and often doubtful, for they attack the vital organs.

In poisoning by strychnia, then, respiration must be kept up by every means, by inspirations of air or of oxygen, by alternate pressure on the thorax, &c.

Opium, the most prompt and sure agent to counteract the tetanic effects of the strychnia, should be given either by the mouth or the rectum as circumstances may indicate. I would administer without hesitation from thirty to forty drops of Sydenham's laudanum in fifty grammes of water and watch the effects.

I stop here, and will complete this subject I hope at some future period, when I will review the more important points.

