# Correction

**Published:** 1847-10

**Authors:** 


### CORRECTION.

The last number of the Examiner contained an elaborate article on the principal antidotes or counter poisons, without the proper credit. It was translated by a young friend for the Examiner, from the *Annuaire de Thérapeutique* of Bouchardat, for 1847.

